# Making the Invisible Visible: Colorimetric and Spectroscopic Detection of Colorless Liquids via Solvatochromic Glass Surfaces

**DOI:** 10.1021/acsomega.5c10492

**Published:** 2026-03-06

**Authors:** Tereza Navrátilová, Martin Havlík, Ameneh Tatar, Ladislav Fišer, Bohumil Dolenský

**Affiliations:** a Department of Analytical Chemistry, 52735 University of Chemistry and Technology, Prague, Technická 5, Praha 166 28, Czech Republic; b Department of Physics and Measurements, 52735 University of Chemistry and Technology, Prague, Technická 5, Praha 166 28, Czech Republic

## Abstract

Absorption spectrophotometry is a reliable, efficient, and widely used analytical technique across scientific and industrial fields; however, its applicability becomes limited when the analyte lacks a visible-region chromophore and therefore appears colorless. To extend its applicability to colorless substances, glass surfaces functionalized with a solvatochromic dye were prepared and spectroscopically evaluated. This study demonstrates, for the first time, the use of glass covalently modified with a stilbazolium-based solvatochromic dye as a robust and reusable transducer for the selective detection and discrimination of colorless organic solvents. The stilbazolium-based dye, structurally derived from Brooker’s merocyanine, was covalently bound to the surface of glass beads and slides via a triethoxysilyl group. The resulting materials exhibited distinct changes in their UV/vis absorption spectra, depending on the solvent, manifested as shifts in absorption band positions and variations in intensity. Unique spectral signatures for more than 20 different solvents, including structurally similar compounds, were successfully resolved, as demonstrated by principal component analysis. Compared to previous approaches using solution-phase indicators or derivatization, this method offers a nondestructive, real-time, and reusable platform for chemical sensing. These changes enabled not only the visual color discrimination of colorless solvents but also their qualitative analysis by using spectrophotometry without the need for derivatization.

## Graphical abstract

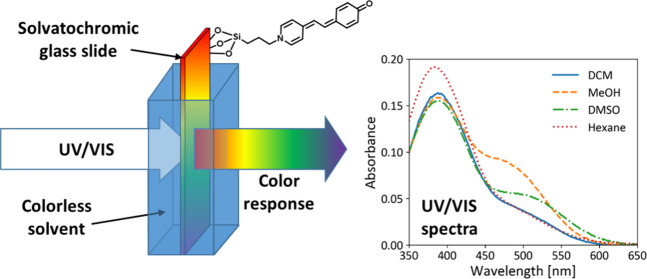

## 1 Introduction

Absorption spectrophotometry (UV/vis) is a versatile analytical technique with applications across numerous fields. However, its usability is limited when the target substance is colorless, as absorption in the UV region can overlap with that of the required solvent, such as acetonitrile, acetone, or toluene. In contrast, absorption in the visible region would enable straightforward detection by the naked eye. Indeed, many important compounds are colorless, including a wide range of organic solvents, such as aliphatic alcohols, saturated hydrocarbons, ethers, aldehydes, ketones, carboxylic acids, and their solutions.

In some cases, colorless compounds can be detected by UV/vis spectrophotometry after derivatization, a process in which a chromophore is irreversibly introduced into the molecule through covalent chemical modification to enable absorption in the UV or visible region.^1–3^ Common reagents for derivatization in high-performance liquid chromatography (HPLC) include ninhydrin, benzoyl chloride (Scheme 1a), and phenyl isocyanate, which facilitate UV/vis detection. However, because derivatization permanently alters the chemical structure of the analyte, this approach is inherently destructive, which limits its applicability in situations where the preservation of the original analyte is required.

**Scheme 1:**
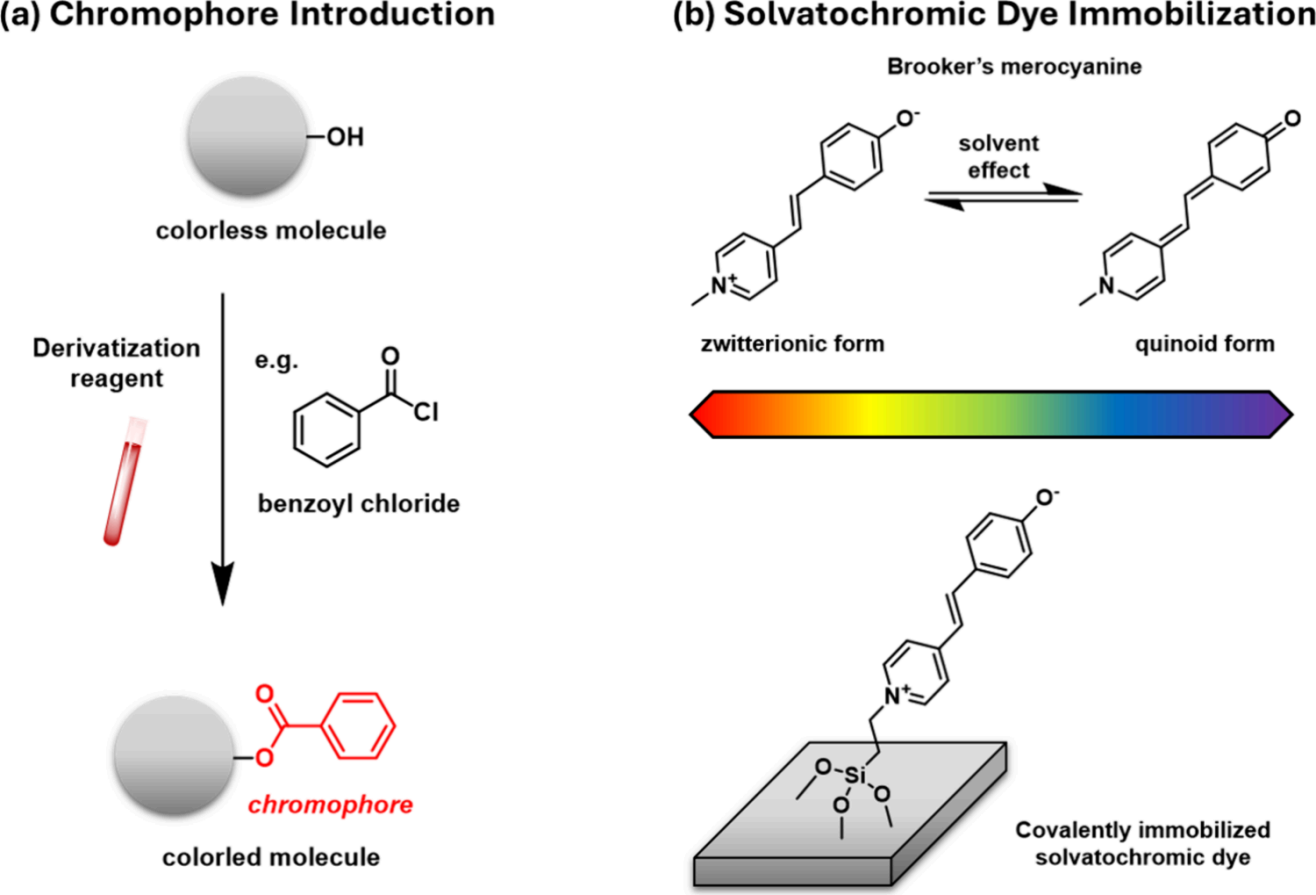
(a) Schematic Derivatization of a Colorless Alcohol with Benzoyl Chloride, Introducing a Chromophore into the Molecule; (b) Solvatochromic Behavior of Brooker’s Merocyanine: Color Change Associated with Its Zwitterionic and Quinonoid Forms and Covalent Immobilization onto a Solid Surface via an Alkyl-Silane Linker

An alternative approach would involve the use of solvatochromic indicators – compounds that exhibit significant color changes in response to the polarity of their environment. These molecules, such as Brooker’s merocyanine, rely on intramolecular charge transfer (ICT) to produce their characteristic optical response. Upon excitation, the electron density shifts from an electron-donating group to an electron-accepting group within the molecule, generating a polarized excited state. Brooker’s merocyanine exists in equilibrium between a neutral quinone-like form and a zwitterionic form, and the relative population of these forms is strongly influenced by the polarity of the surrounding medium. Polar solvents stabilize the zwitterionic form due to solvation of its separated charges, leading to a red-shift in absorption, whereas nonpolar solvents favor the neutral quinone form, resulting in a blue-shifted absorption. This interplay between solvent polarity, ICT, and tautomeric equilibrium enables the sensitive detection of environmental changes, making solvatochromic indicators useful for sensing applications.^4–6^

However, the practical application of such indicators is often limited by the fact that their use typically consumes a portion of the solvatochromic compound during measurement. This consumption leads to contamination of the analyte and gradual degradation of the indicator itself, reducing the reproducibility and sensitivity over time. Consequently, while solvatochromic compounds offer a powerful means for indirect detection of colorless analytes via UV/vis spectrophotometry, careful consideration of indicator stability and solvent interactions is essential for reliable implementation in analytical systems.

To address these limitations, efforts have been made to immobilize solvatochromic dyes onto solid surfaces, either noncovalently or covalently.^7–19^ A covalent attachment is particularly advantageous, as it ensures a stable, insoluble, reusable surface that exhibits consistent color changes without the loss of the solvatochromic compound, which can occur with noncovalent binding. Such surfaces can be easily dried, washed, and reused, offering a cost-effective, rapid response without the need for complex instrumentation.^7,8^

Several covalently modified solvatochromic materials have been reported, including polymers,^9–13^ fibers,^14^ resins,^15^ and mesoporous silicas.^7,16–19^ Silica-based surfaces, in particular, offer significant advantages due to their mechanical strength, chemical inertness, and thermal stability. Unlike most polymers, silica does not swell in organic solvents, making it a robust material for such applications. In previous studies, we demonstrated the use of solvatochromic compounds on silica nanofibers^14^ and porous silicas,^7^ enabling visual discrimination of solvents with different polarities. However, these studies were limited to visual observation of color changes.

In this study, we focused on the modification of glass surfaces with a triethoxysilyl derivative of Brooker’s merocyanine (Scheme 1b), a widely used solvatochromic dye that exhibits color shifts of up to 137 nm when transitioning from chloroform to methanol.^20^ Our primary objective was to develop solvatochromic glass surfaces that allow color changes to be monitored not only visually but also qualitatively using UV/vis spectrophotometry. Specifically, we investigated the modification of glass beads and slides, aiming to demonstrate their potential as functional parts of colorimetric sensors, even for colorless analytes. The spectrophotometric evaluation of solvatochromic responses generates large sets of highly correlated UV/vis spectral data for which conventional univariate analysis may be insufficient. Therefore, principal component analysis (PCA) was employed as a multivariate statistical tool to reduce data dimensionality and to facilitate the discrimination of solvents based on their overall spectral response.

## 2 Experimental Section

### 2.1 Chemicals and Materials

All of the reagents were obtained from commercial suppliers and used without further purification. Glass beads (2 mm diameter, Supelco) were purchased from Merck KGaA (Germany). Standard glass microscope slides (article no. 02 1102; approximately 76 × 26 × 1 mm, precleaned with a frosted end) were acquired from Menzel Gläser (Germany).

### 2.2 Preparation and Characterization of Compounds

#### Stilbazolium Salt **6**

Stilbene **3** (1.00 g, 4.18 mmol; prepared in the 51% yield according to ref 7) was dissolved in acetonitrile (35 mL) in a 100 mL round-bottom flask and heated to 60 °C. Silane **5** (1.0 mL, 1.3 g, 4.0 mmol; prepared in 94% yield according to ref 21) was then added, and the reaction mixture was stirred at 60 °C for 3 days. Upon completion of the reaction, the solvent was evaporated to dryness. The crude residue was purified by column chromatography on silica gel (isocratic elution, dichloromethane: methanol, 93:7 v/v), yielding stilbazolium salt **6** (1.16 g, 49%) as a yellow powder. ^1^H NMR (500 MHz, DMSO-*d*_6_): δ 8.98–8.90 (2H, m, H11), 8.26–8.20 (2H, m, H10), 8.03 (1H, d, 16.3, H7), 7.83–7.74 (2H, m, H5), 7.51 (1H, d, 16.3, H8), 7.33–7.22 (2H, m, H4), 4.46 (2H, t, 7.2, H12), 3.75 (6H, q, 7.0, H15), 2.30 (3H, s, H1), 2.00–1.90 (2H, m, H13), 1.14 (9H, t, 7.0, H16), 0.60–0.51 (2H, m, H14). ^13^C­{^1^H} NMR (126 MHz, DMSO-*d*_6_): δ 169.05 (C2), 152.77 (C9), 151.96 (C3), 144.31 (C11), 139.81 (C7), 132.82 (C6), 129.31 (C5), 123.92 (C10), 123.42 (C8), 122.67 (C4), 61.87 (C12), 57.88 (C15), 24.70 (C13), 20.89 (C1), 18.17 (C16), 6.59 (C14). The spectra are provided in the Supporting Information – SI (Figures S12–S16). HRMS (ESI^+^, MeOH): *m*/*z* calculated for C_24_H_34_NO_5_Si [M]^+^ 444.2206, found 444.2202.

### 2.3 General Procedure for Glass Surface Modification

Commercial glass materials (i.e., untreated beads and slides) are referred to as unmodified in the following text. These were first cleaned by immersion in a 1:1 v/v mixture of methanol and aqueous hydrochloric acid (36%) for 3 h at room temperature, then rinsed thoroughly with distilled water, and dried in a laboratory oven at 110 °C for 3 h. To obtain blank glass materials (i.e., activated but dye-free surfaces), three surface activation protocols were tested, based on standard literature methods:^22,23^ (A) treatment with 1:1 methanol/hydrochloric acid (36%), (B) sulfuric acid (98%), and (C) piranha solution, prepared by mixing three parts sulfuric acid (98%) with one part hydrogen peroxide (30%). In all cases, activation was carried out at room temperature for 3 h followed by rinsing with distilled water and drying at 110 °C for 5 h.

To prepare modified glass materials (i.e., functionalized with solvatochromic dye), the activated glass materials were immersed in an ethanolic solution of stilbazolium salt **6** (25 mmol/L), with a small amount of acetic acid (ethanol:acetic acid, 200:1 v/v) added to catalyze the silanization reaction. The reaction was carried out overnight at 70 °C. After surface functionalization, the materials were treated with a saturated aqueous solution of potassium carbonate for 5 min at room temperature to neutralize residual acid and convert the surface-bound stilbazolium salt into its solvatochromic form. Finally, the materials were washed with UV/vis-grade ethanol and dried at 110 °C for 3 h.

### 2.4 UV/vis Spectrophotometry of Beads

UV/vis spectra of the modified glass beads were measured at ambient temperature. For each measurement, approximately 50 beads were placed in a vertically oriented glass tube (inner diameter of 5 mm) sealed at the bottom, forming a pad 6 cm in height. Measurements were conducted with either dry beads or beads immersed in the solvent under investigation. To minimize ambient light interference, the tube was wrapped in aluminum foil and further covered with heavy-weight paper. Illumination was provided from the top using a Volpi Intralux 6000–1 light source (Artisan Technology Group), which emits high-intensity cold halogen light via a 75 cm fiber optic cable (13 mm outer diameter and 8 mm inner diameter). A second optical fiber was attached to the bottom of the tube to transmit light to the spectrometer. Spectra were recorded using a Red Tide USB650 Fiber Optic Spectrometer (Ocean Optics) equipped with a Sony ILX511 linear silicon CCD array detector. Light transmission to the detector was achieved via a QP400–1-UV–vis optical fiber (Ocean Optics) with a 400 μm core diameter and 1.2 m length. Spectra were collected over the 350–1000 nm range, with 651 data points per spectrum, integration time of 0.2 s, and single scan per measurement. No background correction or instrument calibration was applied. Reference measurements (blank spectra, dye-free) were obtained by using the activated beads. Final spectra were obtained by subtracting the corresponding blank spectra from those of the modified beads. All measured spectra were normalized to 650 nm, a wavelength at which the compound exhibits no absorption, to correct for fluctuations in the light source intensity. A schematic representation of the measurement setup is provided in the SI (Figure S1).

### 2.5 UV/vis Spectrophotometry of Slides

UV/vis spectra of the glass slides were recorded at ambient temperature using a Cary 60 UV–Vis spectrophotometer (Agilent Technologies). For each measurement, a slide was positioned vertically, with its frosted end face perpendicular to the instrument’s light beam path (defining the *z*-axis). Spectra were collected over the range of 190–1100 nm using a scan rate of 4800 nm/min, data interval of 1 nm, and averaging time of 0.0125 s. To improve measurement reproducibility, three scans were recorded for each sample at slightly varied positions along the *xy*-plane (relative to the beam path), and the spectra were averaged. For measurements involving solvent exposure, the slide was immersed in solvent enclosed within a sealed, clear low-density polyethylene (LDPE) foil pouch, ensuring that no air bubbles were present. This setup served both to prevent solvent evaporation and to protect the spectrometer hardware from potential damage caused by volatile or aggressive solvents. The spectra of the modified slide (activation method B) recorded under these conditions were corrected by subtracting a reference spectrum of a blank slide (activation method B, dye-free), enclosed in an identical LDPE foil pouch containing an identical solvent, thereby accounting for the contributions of both the foil and the dry substrate. A schematic representation of the measurement setup is provided in the SI (Figure S2). Control UV/vis measurements of the solvent before and after contact with the functionalized surface confirmed negligible dye leaching, highlighting the benefit of covalent immobilization.

### 2.6 Principal Component Analysis

Principal component analysis (PCA) was applied to UV/vis spectra recorded for a modified glass slide (activation method B) immersed in 22 different colorless solvents. For each solvent, the final spectrum was obtained by averaging three independent measurements taken at different lateral positions on the slide to minimize the impact of spatial variability. The averaged spectra were corrected by subtracting the corresponding averaged spectra of the blank slide (activation method B, dye-free) and baseline-adjusted (intensity at 700 nm set to zero) to eliminate systematic offsets. Spectra were then trimmed to the 340–700 nm region to exclude solvent absorption edges and retain only the informative portion of the signal, yielding 361 variables (wavelengths) per sample. Prior to PCA, all spectral data were mean-centered and scaled to unit variance, thereby standardizing them and ensuring equal contributions of all wavelengths. PCA was conducted on the covariance matrix of the standardized data to reduce the dimensionality and identify the main directions of spectral variation. The proportion of the total variance explained by each principal component was calculated to evaluate the effectiveness of the dimensionality reduction.

## 3 Results and Discussion

### 3.1 Preparation of Compounds

Based on our previous work, we decided to prepare a stilbazolium salt with a triethoxysilane moiety instead of a trimethoxysilane group, as the latter exhibited excessive reactivity (low stability), making subsequent modifications and purification challenging.^7^ Triethoxysilane was selected due to its lower reactivity compared to trimethoxysilane while still providing sufficient hydrolytic activity for effective surface grafting.

Stilbazolium salt **6** was prepared via a three-step synthesis (Scheme 2). The first step was the aldol condensation of 4-methylpyridine (**1**) and 4-hydroxybenzaldehyde (**2**) to give stilbene **3** in a 51% yield.^7^ In the next step, stilbene **3** was *N*-alkylated with triethoxy­(3-iodopropyl)­silane (**5**) to give stilbazolium salt **6** in 49% yield. The silane **5** used for the alkylation was freshly prepared by the Finkelstein reaction from the corresponding (3-chloropropyl)­triethoxysilane (**4**) in a 94% yield.^21^

**Scheme 2:**
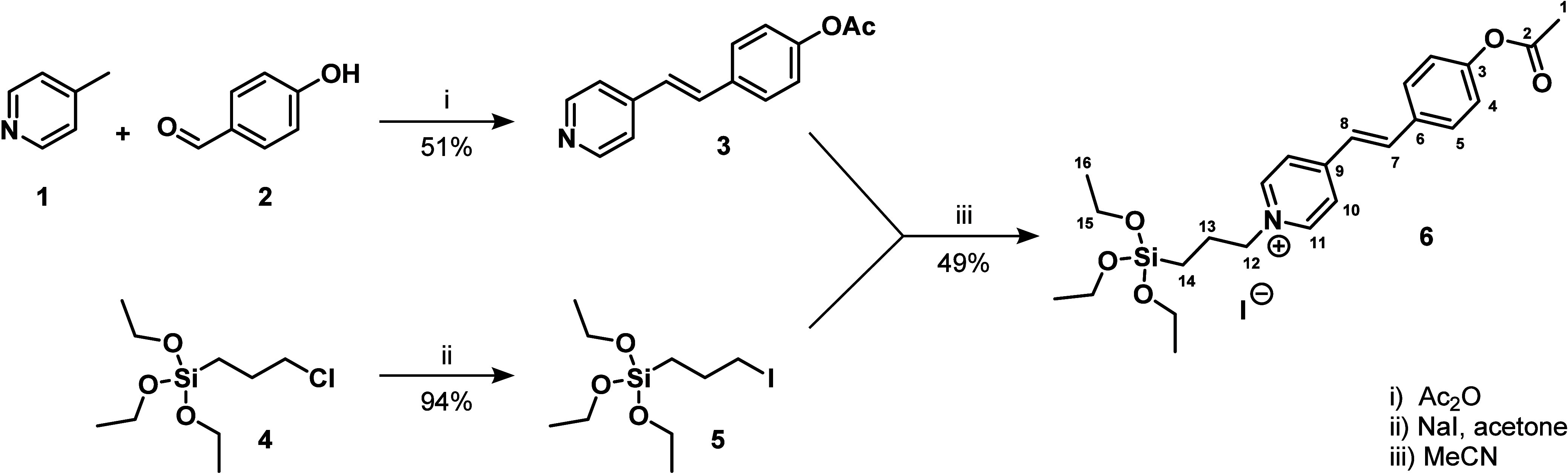
Preparation of Stilbazolium Salt **6** Bearing a Silane Moiety for Covalent Modification of Activated Glass Surfaces^a^

a The skeletal numbering attributed to salt **6** corresponds to the assignment of the NMR signals.

To activate the solvatochromic behavior of stilbazolium salt **6**, removal of the acyl group was necessary. Our attempts to deacetylate salt **6** using potassium carbonate yielded the solvatochromic neutral form; however, the silane moiety proved to be unstable. This instability was confirmed by HRMS and NMR spectroscopy; see the SI (Figures S17–S22). It is likely that under basic or protic conditions, the silane moiety undergoes nucleophilic attack, leading to hydrolysis and alcohol exchange reactions. Consequently, we decided to postpone deacetylation to the next step, i.e., after binding stilbazolium salt **6** to a glass surface.

### 3.2 Covalent Modification of Glass Surfaces

First, the commercial beads (or slides), termed unmodified materials, were cleaned by immersion in a methanol/hydrochloric acid mixture. The surfaces were then activated using standard activation methods^22,23^ to produce the blank materials (dye-free). In the case of the slides, three activation methods were examined: (A) methanol/hydrochloric acid, (B) H_2_SO_4_, and (C) piranha solution, each followed by thorough washing and drying to obtain activated blank materials. Surface activation was necessary to generate free silanol groups on the glass, providing reactive sites for the subsequent covalent attachment of the silane moiety. For the beads, only the H_2_SO_4_ method (B) was used, as it was found to be the most suitable in prior experiments on glass slides. Although piranha solution is highly effective in maximizing surface hydroxylation, it is extremely aggressive and difficult to remove completely due to its high viscosity and poses safety risks, so its use was limited to initial tests on slides.

Subsequent modification (Scheme 3) was carried out by heating the activated blank materials in an ethanolic solution of stilbazolium salt **6** with a catalytic amount of acetic acid. Acetic acid served to catalyze the hydrolysis of the ethoxy groups on the silane moiety, promoting the formation of reactive silanol groups that could then condense with the hydroxyl groups on the glass surface to form stable Si–O–Si bonds. After the covalent attachment, the materials were treated with an aqueous solution of potassium carbonate to remove the acetyl group, thus restoring the solvatochromic response. Final washing and drying provided the modified materials, i.e., the solvatochromic beads or slides.

**Scheme 3:**
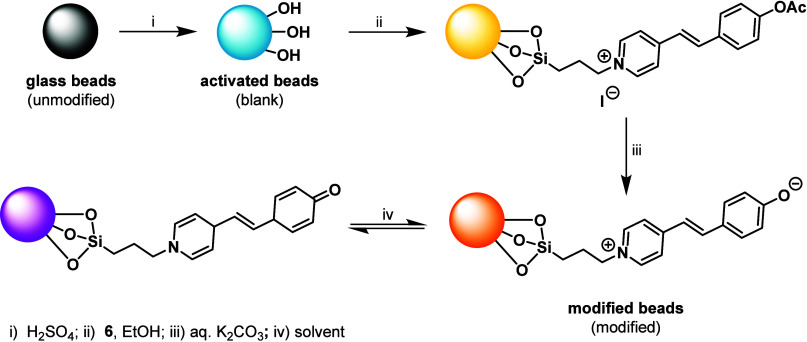
Modification of Glass Surfaces with Solvatochromic Stilbazolium Dye **6**

### 3.3 UV/vis Response of Modified Surfaces

#### 3.3.1 UV/vis Response of Modified Beads

The UV/vis response of the modified beads was compared to that of blank beads (dye-free). The modification was clearly observable to the naked eye, with the blank beads being colorless, while the modified beads appeared yellowish when dry. Portions of the beads were then immersed in methanol (MeOH), ethanol (EtOH), dimethylformamide (DMF), and dimethyl sulfoxide (DMSO) individually. A slight color change was visible to the naked eye, although it was difficult to capture this subtle change on a digital camera. Fortunately, the color change was subsequently recorded qualitatively using UV/vis spectrophotometry (see Figure 1).

**Figure 1:**
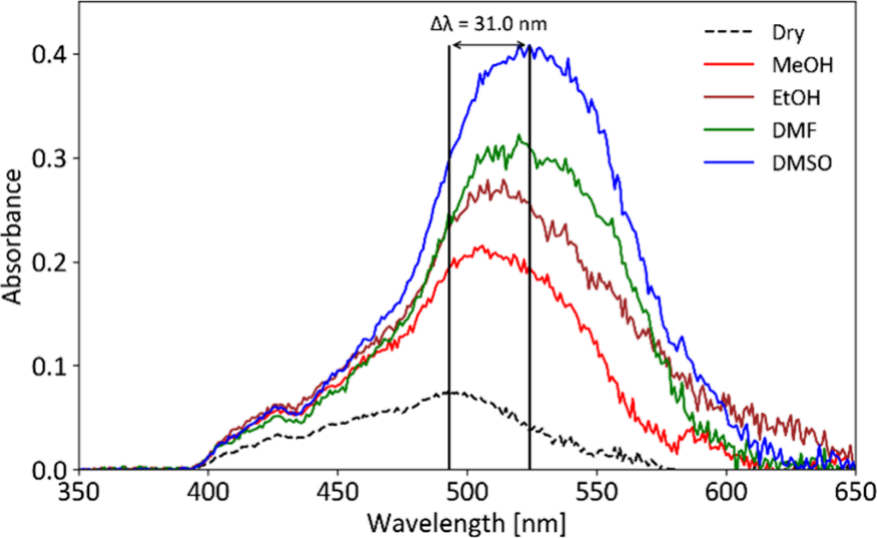
Solvatochromic response of dry modified beads (dashed line) and beads immersed in solvents of varying polarity, as studied by UV/vis spectrophotometry.

The recorded spectra confirmed that the modified beads exhibited a clear solvatochromic response, with significant changes in both the position and intensity of the absorption band in the 400–650 nm range. The maximum absorption wavelength (λ_max_) for the dry modified beads was 493 nm. In solvents, λ_max_ values were 506 nm in MeOH, 508 nm in EtOH, 520 nm in DMF, and 524 nm in DMSO. This trend is consistent with the behavior of merocyanine-based solvatochromic dyes, such as Brooker’s merocyanine, which shows λ_max_ values of 490 nm in MeOH and 572 nm in DMSO.^20^ As expected, the absorption maximum shifts to longer wavelengths (31 nm red-shift) as the polarity of the solvent decreases.^7,20^

### 3.4 UV/vis Response of Modified Slides

We next studied the UV/vis response of the modified slides. First, we evaluated the effect of the activation method by comparing UV/vis spectra of the unmodified slide, blank slides (dye-free) activated by (A) MeOH/hydrochloric acid, (B) H_2_SO_4_, and (C) piranha solution, and the corresponding modified slides. As shown in Figure 2, the activation method had little impact on the UV/vis spectra of the blank slides, but significant differences emerged after modification. All modified slides exhibited a strong absorption band at 371 nm, attributable to the immobilized stilbazolium dye **6**.^7^ Among them, the slide activated with H_2_SO_4_ showed a more intense absorption at 371 nm. Based on these results, we selected the modified slide prepared from H_2_SO_4_-activated glass (method B) for detailed solvatochromic studies, anticipating higher sensitivity to chemical stimuli.

**Figure 2:**
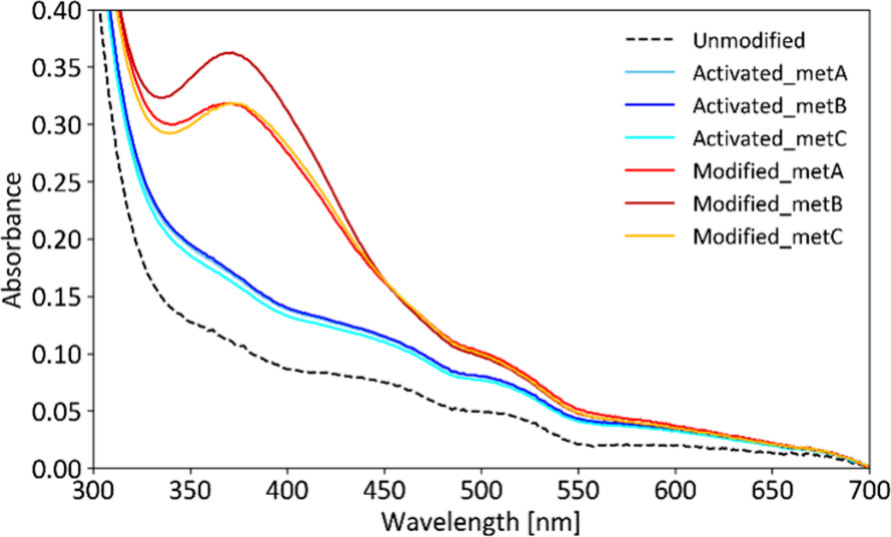
UV/vis spectra of the unmodified slide, slides activated by methods A, B, and C (blank slides, dye-free), and corresponding modified slides.

In addition, we recorded UV/vis spectra of one, two, and three stacked slides to observe changes in the response. As expected, the response gradually increased (SI, Figure S3).

The solvatochromic response of the modified slide (activation method B) was examined by immersing it individually in 22 different solvents; the spectra of all tested solvents are provided in the SI (Figures S4–S6). Distinct color changes in shades of orange, pink, and purple were visible to the naked eye, however difficult to capture with a digital camera. In contrast, spectrophotometry clearly revealed unique spectral signatures for each solvent in the visible region. Notably, even structurally similar compounds, such as primary alcohols, produced distinguishable spectra.

In contrast to the modified beads immersed in DMSO (Figure 1), which exhibit only a band at 524 nm, the modified slide immersed in DMSO (Figure 3) exhibits two absorption maxima at 380 and 510 nm. This is comparable to Brooker’s merocyanine, which, in DMSO solution, exhibits two absorption maxima at 395 and 581 nm.^7^ The difference in absorption maxima positions and intensities can be attributed to the distinct molecular environments of the dye in solution and when immobilized on the glass surface, where surface hydroxyl groups and local morphology likely influence the electronic properties of the bound chromophore.^24^

**Figure 3:**
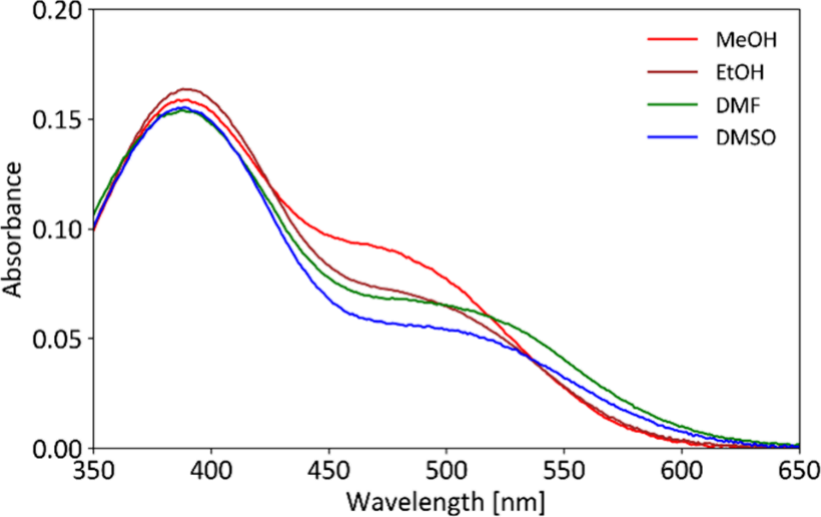
Blank corrected UV/vis spectra of the modified slide (activation method B) immersed in solvents of varying polarity, as studied by UV/vis spectrophotometry.

### 3.5 Resolving Solvatochromic Behavior through Principal Component Analysis

Given the subtle spectral differences observed for closely related solvents, we recognized that a simple visual or manual comparison of the spectral data is insufficient to capture the changes in detail. Therefore, to better interpret the complex solvent-induced variations and uncover potential patterns or clustering in the data, we applied PCA as a multivariate statistical tool.

The eigenvalues obtained by PCA revealed that the first three principal components (PC1–PC3) accounted for 90.2% of the total variance in the data set (SI, Figure S7), indicating that most of the spectral variability could be effectively represented in a reduced-dimensional space. PC1–PC3 correspond to orthogonal linear combinations of the original UV/vis spectral variables that capture the largest variance in the data set; in total, 20 principal components were obtained, with higher-order components contributing only marginally to the remaining variance. The PCA output was visualized in two- (SI, Figures S8–S10) and three-dimensional scatter plots (Figure 4), which demonstrated that the solvatochromic slide could distinguish between all 22 tested solvents, listed according to the E_T_(30) scale, a Dimroth–Reichardt solvent polarity parameter that ranks solvents based on their polarity.^4^

**Figure 4:**
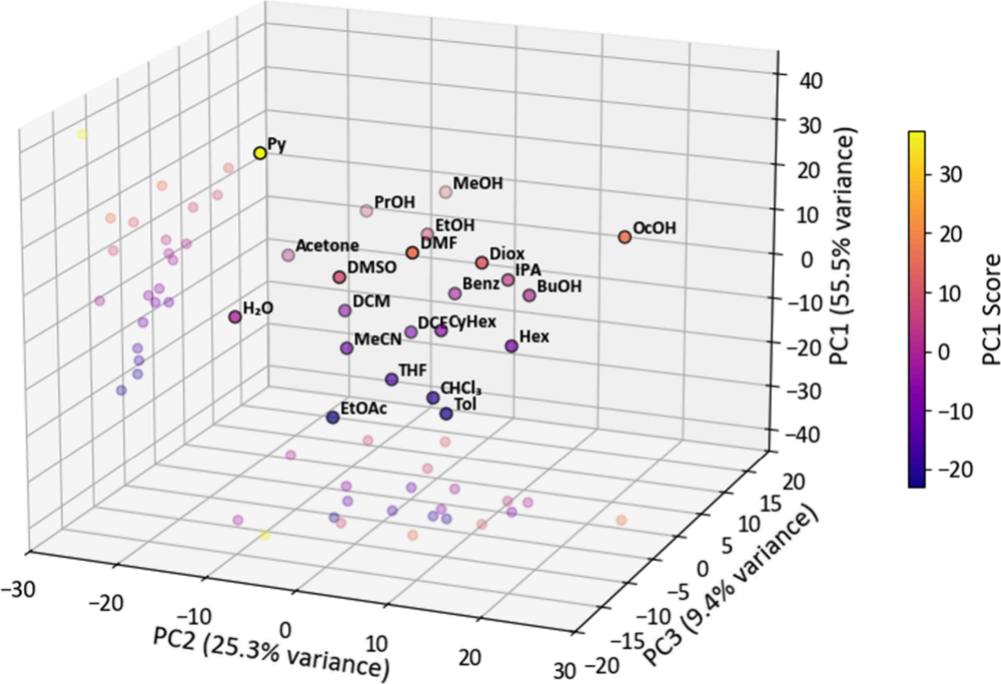
Principal component analysis (PCA) of the UV/vis spectra of the modified slide immersed in 22 different solvents. The 3D score plot shows the distribution along PC1–PC2–PC3, with projections onto the PC1–PC3 and PC2–PC3 planes to aid visualization. Solvents are sorted by increasing E_T_(30) values and labeled in brackets with their abbreviations and E_T_(30) values in kcal·mol^–1^ [4]: Cyclohexane (CyHex, 30.9), Hexane (Hex, 31.0), Toluene (Tol, 33.9), Benzene (Benz, 34.3), 1,4-Dioxane (Diox, 36.0), Tetrahydrofuran (THF, 37.4), Ethyl acetate (EtOAc, 38.1), Chloroform (CHCl_3_, 39.1), Pyridine (Py, 40.5), Dichloromethane (DCM, 40.7), 1,2-Dichloroethane (DCE, 41.3), Acetone (Acetone, 42.2), *N*,*N*-Dimethylformamide (DMF, 43.2), Dimethylsulfoxide (DMSO, 45.1), Acetonitrile (MeCN, 45.6), Octan-1-ol (OcOH, 48.1), Isopropanol (IPA, 48.4), Butan-1-ol (BuOH, 49.7), Propan-1-ol (PrOH, 50.7), Ethanol (EtOH, 51.9), Methanol (MeOH, 55.4), Water (H_2_O, 63.1). Clustering reflects solvent-induced spectral variations with color coding by PC1 scores to highlight dominant trends.

Clustering patterns in the PCA scores reflect solvent families and their dominant physicochemical properties. Nonpolar hydrocarbons (hexane, cyclohexane, benzene, and toluene) grouped closely, as did halogenated solvents (dichloromethane, chloroform, and 1,2-dichloroethane) and ethers (tetrahydrofuran and 1,4-dioxane). Primary alcohols (methanol, ethanol, propan-1-ol, butan-1-ol, and octan-1-ol) formed a coherent group, with separation along PC3 highlighting differences in chain length and branching, exemplified by the distinct position of isopropyl alcohol and the amphiphilic character of octanol. Strongly polar aprotic solvents (acetonitrile, *N*,*N*-dimethylformamide, and dimethylsulfoxide) clustered together, while water and acetone appeared nearby yet remained clearly separated from the alcohols, reflecting their high polarity and strong hydrogen-bonding interactions. Pyridine emerged as an isolated outlier, consistent with its unique combination of polarity and basicity, which strongly perturbs the solvatochromic response of the modified slide.

Notably, the PCA resolved subtle differences between structurally similar substances, underlining the method’s sensitivity. For example, toluene and benzene are close in E_T_(30) (33.9 and 34.3, respectively; difference of 0.4, i.e., + 1.2%), yet they are well distinguished by PC1. Similarly, cyclohexane and hexane (30.9 and 31.0, respectively; difference of 0.1, i.e., + 0.3%) are well distinguished by PC2. In contrast, toluene and chloroform differ significantly in both molecular structure and E_T_(30) (33.9 and 39.1, respectively; difference of 5.2, i.e., + 15.3%) but appear closer in the PC1–PC3 space. This likely arises because our slide uses a dye different from that employed in the determination of the E_T_(30) values, indicating that the solvatochromic response depends on both solvent polarity and specific molecular interactions with the dye. Incorporating additional slides with dyes exhibiting uncorrelated solvatochromic responses is expected to further enhance the robustness and fidelity of solvent recognition.

## 4 Conclusions

This foundational study demonstrates that a solvatochromic dye can be covalently immobilized onto glass substrates via a triethoxysilane linker while retaining its characteristic solvatochromic responsiveness. The resulting functionalized glass slides and beads both exhibit solvent-dependent spectral changes; however, the planar glass slides provide more distinct, reproducible, and readily interpretable UV/vis responses across a broad range of colorless solvents, including those absorbing exclusively in the UV region. In contrast, the bead-based substrates display only a single broad absorption feature, limiting their analytical discrimination capability. Notably, the slide-based approach enables straightforward differentiation of even structurally similar or isomeric solvents based solely on their spectral signatures, surpassing the performance of solution-phase indicators and noncovalently immobilized dyes.

Principal component analysis (PCA) proved to be a powerful tool for visualizing the complex, multivariate spectral responses of the surface-bound solvatochromic dye. PCA revealed clear solvent-dependent trends and confirmed that a single modified slide is sufficient to discriminate among more than 20 chemically related solvents. Importantly, the spectral response of the functionalized substrates remained stable over repeated measurement cycles during this study, indicating good short-term reproducibility and supporting the robustness of the covalent immobilization strategy. The effects of long-term exposure and extended repeated use represent important topics for future investigation.

Beyond the present work, several directions can further strengthen the analytical capabilities of this platform. The application of unsupervised clustering methods, such as k-means analysis applied to PCA score plots, could provide an additional objective assessment of class separation and solvent discrimination. Likewise, blind testing protocols would offer a rigorous demonstration of the predictive power of the combined solvatochromic surface–PCA approach. Extending the methodology to more complex matrices, such as alcoholic beverages or other real-world samples spiked with small amounts of solvent (e.g., methanol in ethanol/water mixtures), would further highlight the practical applicability of this sensing concept.

Overall, these results establish functionalized glass surfaces as a novel class of sensitive, reusable, and nondestructive optical transducers for the direct recognition of colorless liquids. The pronounced, reversible, and visually apparent solvatochromic responses suggest strong potential for both instrumental and naked-eye evaluation, including integration with smartphone-based readout systems. Future sensor arrays incorporating multiple, chemically distinct solvatochromic dyes may further expand this platform toward qualitative and semiquantitative analysis of complex liquid mixtures in real-world applications.

## Supplementary Material



## Notes

Detailed descriptions of the UV/vis spectrophotometry setups, representative UV/vis spectra of the glass beads and slides (both dry and in solvents), additional details on PCA outputs, and the complete set of 1D and 2D NMR spectra of the modified stibazolium dye, together with details of the NMR and HRMS measurements (PDF)
