# Supplementary material for: Making the Invisible Visible: Colorimetric and Spectroscopic Detection of Colorless Liquids via Solvatochromic Glass Surfaces
Source: ACS Omega. 2026 Mar 6;11(10):16026–33. doi: 10.1021/acsomega.5c10492 (PMC13000624; doi:10.1021/acsomega.5c10492)
Supplement: Supplementary file 1 [file ao5c10492_si_001.pdf]

SUPPLEMENTARY INFORMATION

Making the Invisible Visible: Colorimetric and Spectroscopic Detection of Colorless Liquids via Solvatochromic Glass Surfaces

Tereza Navrátilová[a], Martin Havlík[a], Ameneh Tatar[a], Ladislav Fišer[b] and Bohumil Dolenský[a,*]

[a] *Department of Analytical Chemistry, University of Chemistry and Technology, Prague, Technická 5, 166 28 Praha, Czech Republic*

[b] *Department of Physics and Measurements, University of Chemistry and Technology, Prague, Technická 5, 166 28 Praha, Czech Republic*

CONTENTS

1. General S1
   - 1.1 Nuclear Magnetic Resonance Spectroscopy (NMR) S1
   - 1.2 High-Resolution Mass Spectrometry (HRMS) S1
2. UV/VIS spectra of glass beads S1
   - 2.1 Experimental setup and UV/VIS measurement S1
3. UV/VIS spectra of glass slides S2
   - 3.1 Experimental setup and UV/VIS measurement S2
   - 3.2 Dry slides stacking study S2
   - 3.3 UV/VIS spectra of the modified slide immersed in various solvents S3
4. Principal Component Analysis (PCA) S4
   - 4.1 Explained variance plot S4
   - 4.2 2D PCA score plots S5
5. NMR spectra of stilbazolium salt **6** S6
6. Decomposition of deacetylated stilbazolium salt **6** S9
   - 6.1 HRMS S9
   - 6.2 NMR spectra S9

1. General

1.1 Nuclear Magnetic Resonance Spectroscopy (NMR)

The NMR spectra were recorded on a JNM-ECZ500R NMR spectrometer (JEOL Resonance) operating at 500.16 MHz for ^{1}H and 125.77 MHz for ^{13}C. All spectra were referenced to tetramethylsilane using the residual solvent signal of DMSO-d_6 (^{1}H NMR: 2.50 ppm; ^{13}C NMR: 39.52 ppm). The chemical shifts (δ) are given in ppm. The coupling constants (*J*) are given in Hz. Measurements were performed at 25 °C.

1.2 High-Resolution Mass Spectrometry (HRMS)

The HRMS was performed using an LTQ Orbitrap Velos mass spectrometer (Thermo Scientific), a hybrid Ion trap-Orbitrap instrument equipped with an Ion Max ion source and an H-ESI II probe. The conditions for HRMS detection were as follows: ionization mode ESI^{+}; spray voltage 3.0 kV; source temperature 250 °C; capillary temperature 300 °C; FTMS measurement mode; resolution (FWHM) 30,000; lock mass at 413.2662 Da (diisooctyl phthalate); and scan range 150–2000 Da. The samples were injected under direct injection conditions (FIA) using an Accela 600 pump (Thermo Scientific), with a sample dosage of 5 µL (Rheodyne valve injection loop), methanol as the mobile phase, and a flow rate of 150 µL/min.

2. UV/VIS spectra of glass beads

2.1 Experimental setup and UV/VIS measurement

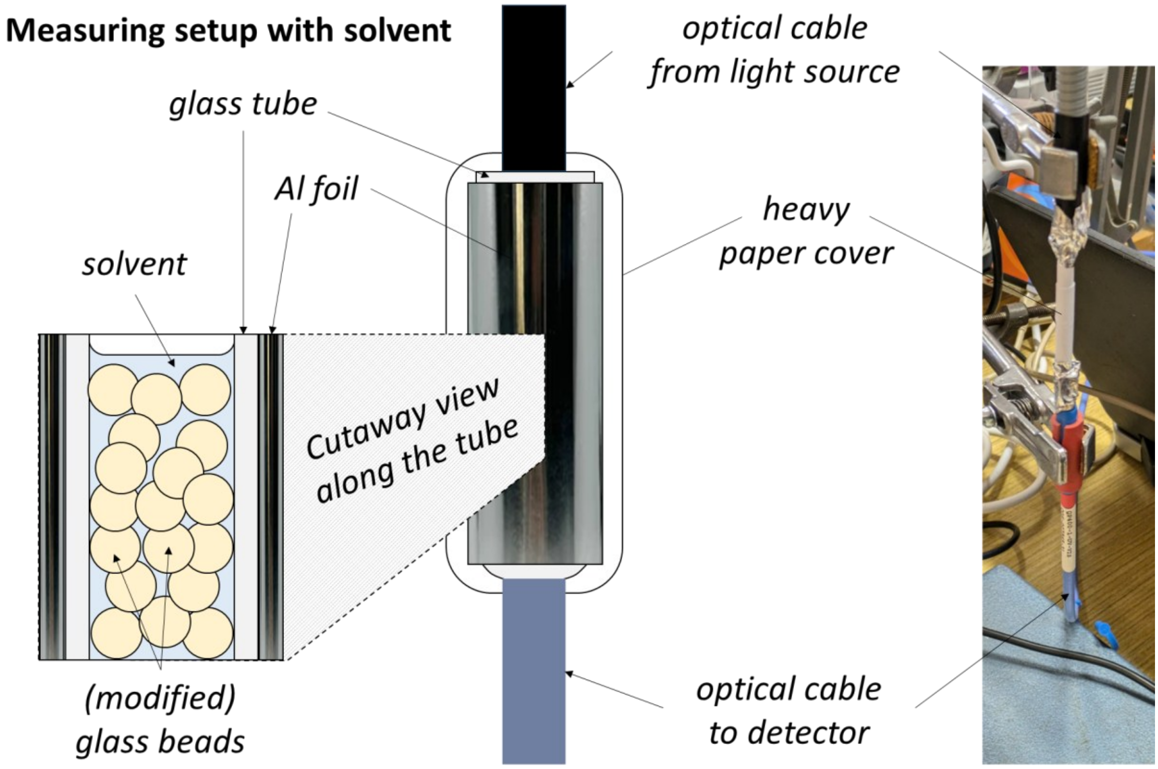

Fig. S1 Schematic representation of the experimental setup used to measure UV/VIS spectra of glass beads.

3. UV/VIS spectra of glass slides

3.1 Experimental setup and UV/VIS measurement

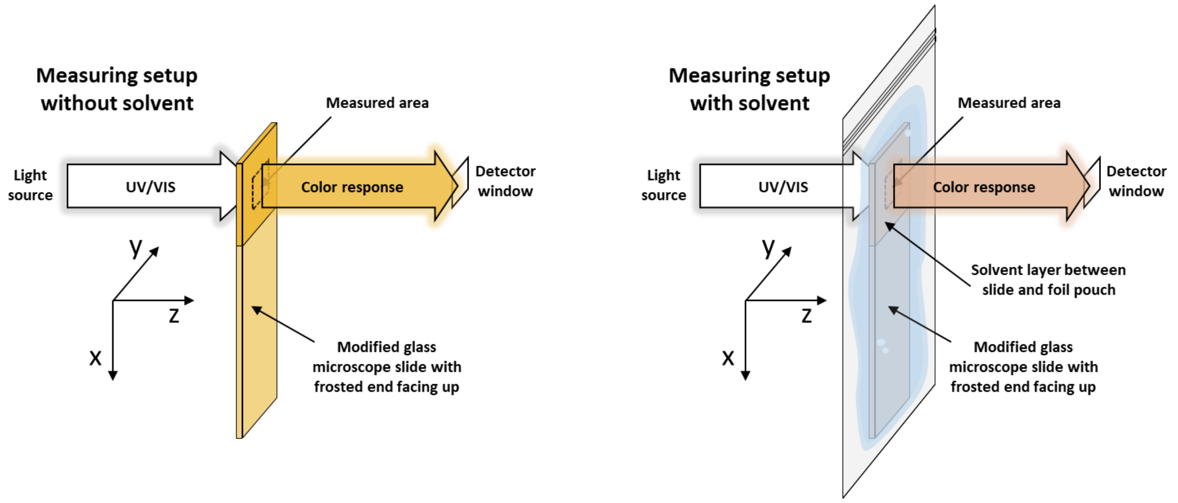

Fig. S2 Schematic representation of the experimental setup for UV/VIS spectrophotometric measurements of glass slides. The figure shows an example setup for a modified slide, which is positioned perpendicular to the spectrophotometer beam within the measuring cell. For measurements in solvent, the slide is immersed in the solvent and enclosed in a sealed LDPE foil pouch. Blank (unmodified) slides were measured using the same setup for reference and background correction.

3.2 Dry slides stacking study

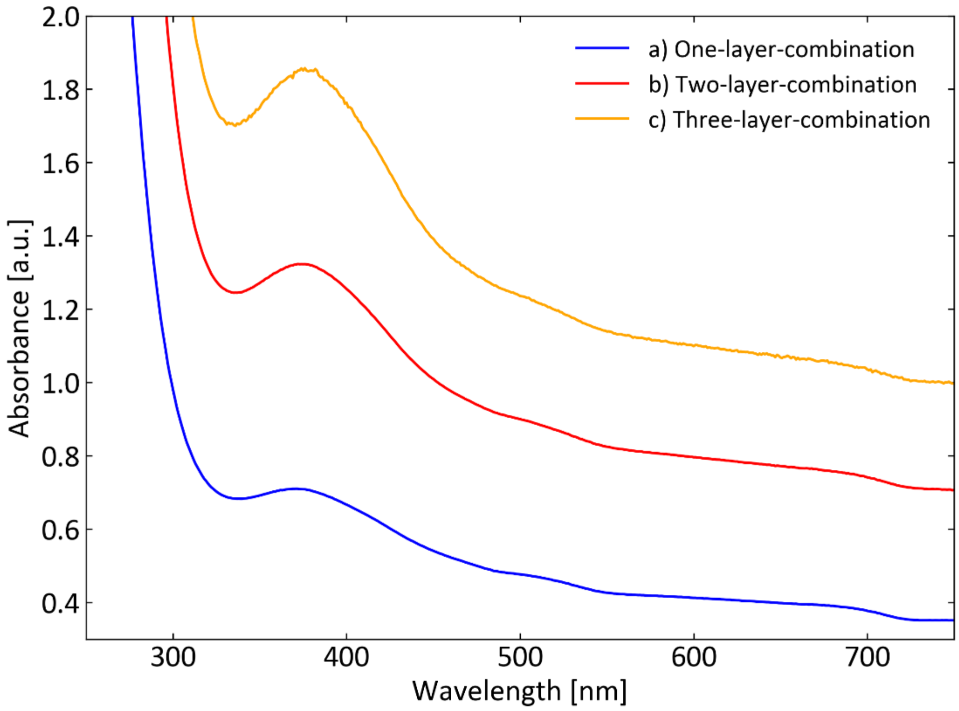

Fig. S3 Comparison of UV/VIS spectra of slide stacks prepared using activation methods A, B and C. **a)** Averaged spectra of individual dry slides (A, B and C). **b)** Averaged spectra of dry slide pairs in both stacking orientations (A-B, B-A, A-C, C-A, B-C, and C-B). **c)** Spectrum of the three-slide stack composed of dry slides A, B and C (A-B-C).

3.3 UV/VIS spectra of the modified slide immersed in various solvents

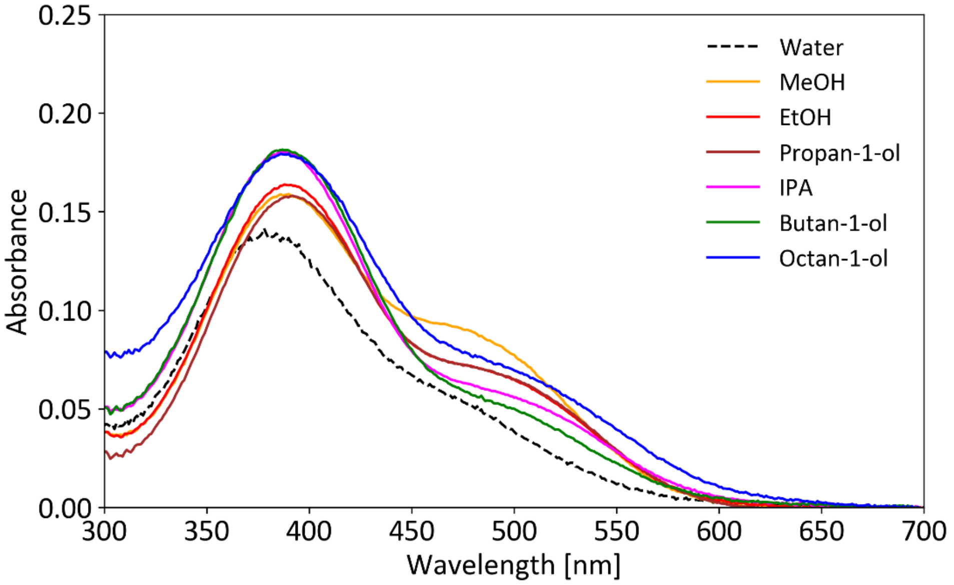

Fig. S4 UV/VIS absorption spectra of the slide modified via method B, measured in water, methanol (MeOH), ethanol (EtOH), propan-1-ol, propan-2-ol, butan-1-ol, and octan-1-ol.

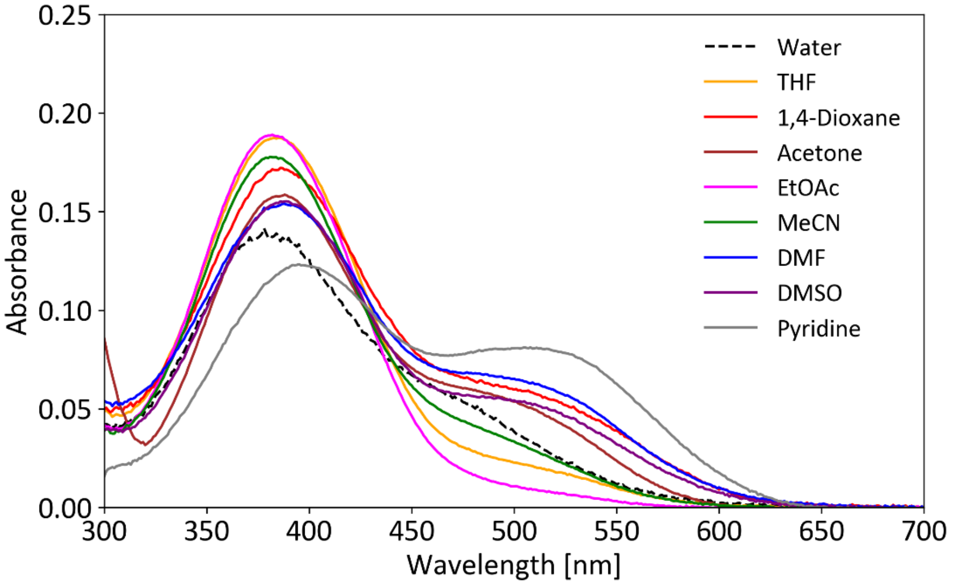

Fig. S5 UV/VIS absorption spectra of the slide modified via method B, measured in water, tetrahydrofuran (THF), 1,4-dioxane, acetone, ethyl acetate (EtOAc), acetonitrile (MeCN), *N*,*N*-dimethylformamide (DMF), dimethyl sulfoxide (DMSO), and pyridine.

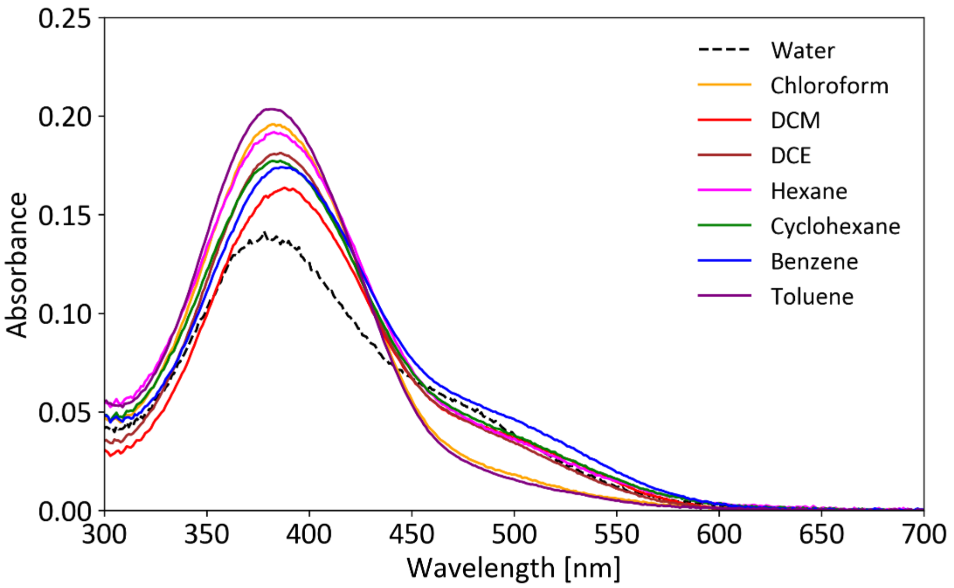

Fig. S6 UV/VIS absorption spectra of the slide modified via method B, measured in water, chloroform, dichloromethane (DCM), 1,2-dichloroethane (DCE), *n*-hexane, cyclohexane, benzene, and toluene.

4. Principal Component Analysis (PCA)

4.1 Explained variance plot

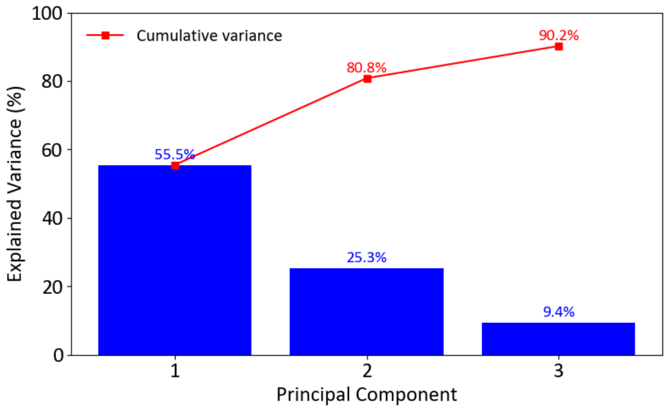

Fig. S7 Explained variance plot for individual principal components and cumulative variance captured by their combination.

4.2 2D PCA score plots

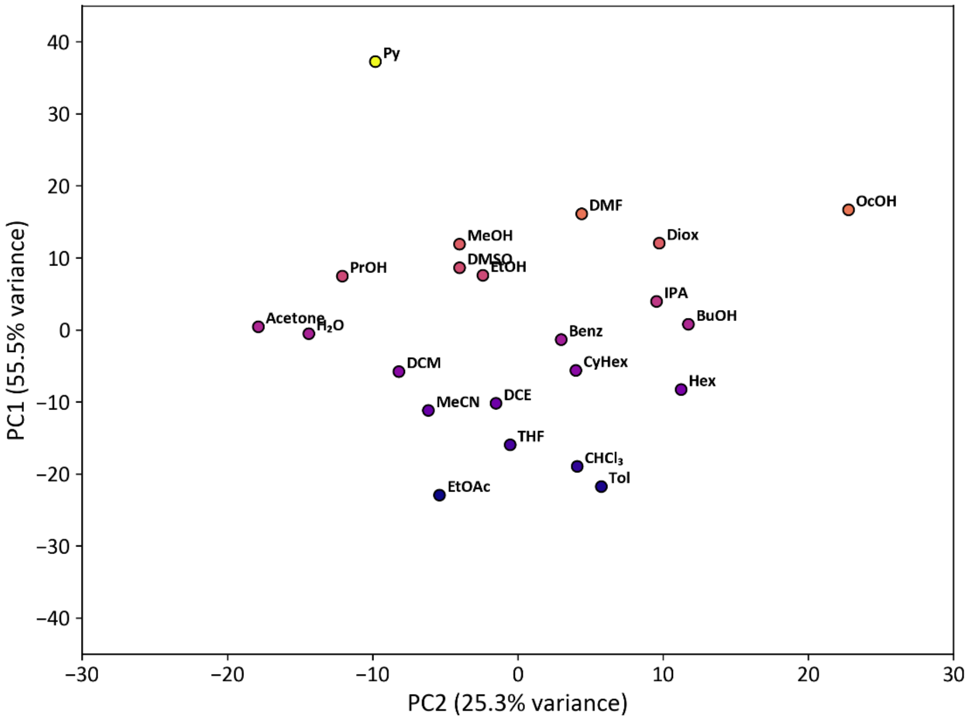

Fig. S8 2D principal component analysis (PCA) score plot of UV/VIS spectra of the modified slide in 22 different solvents, showing PC2 versus PC1. Color coding corresponds to PC1 scores as defined in Figure S7.

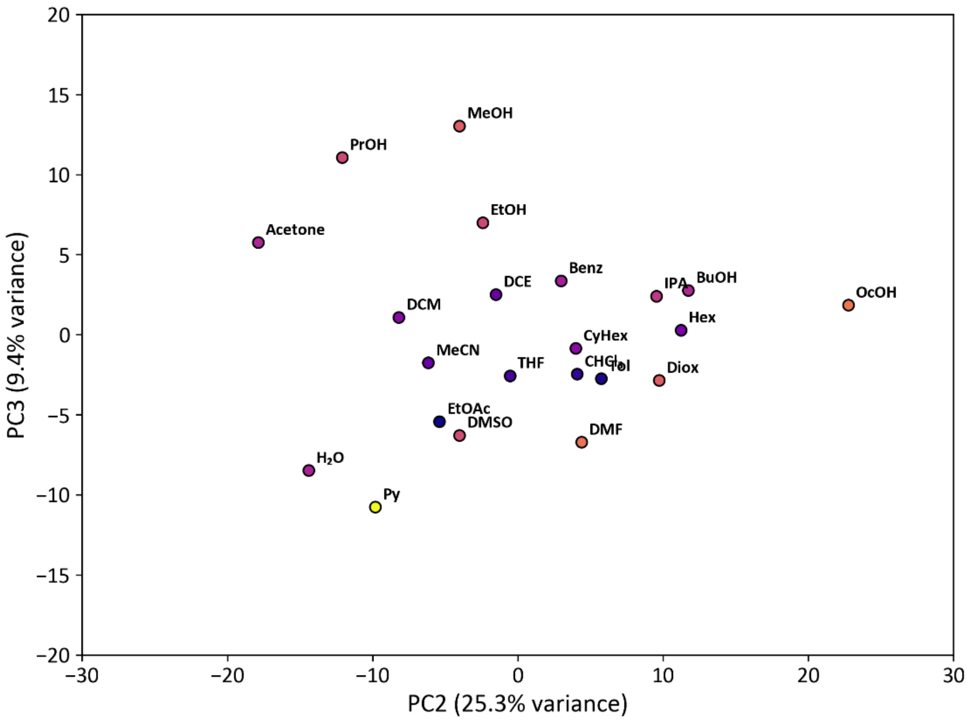

Fig. S9 2D principal component analysis (PCA) score plot of UV/VIS spectra of the modified slide in 22 different solvents, showing PC2 versus PC3. Color coding corresponds to PC1 scores as defined in Figure S7.

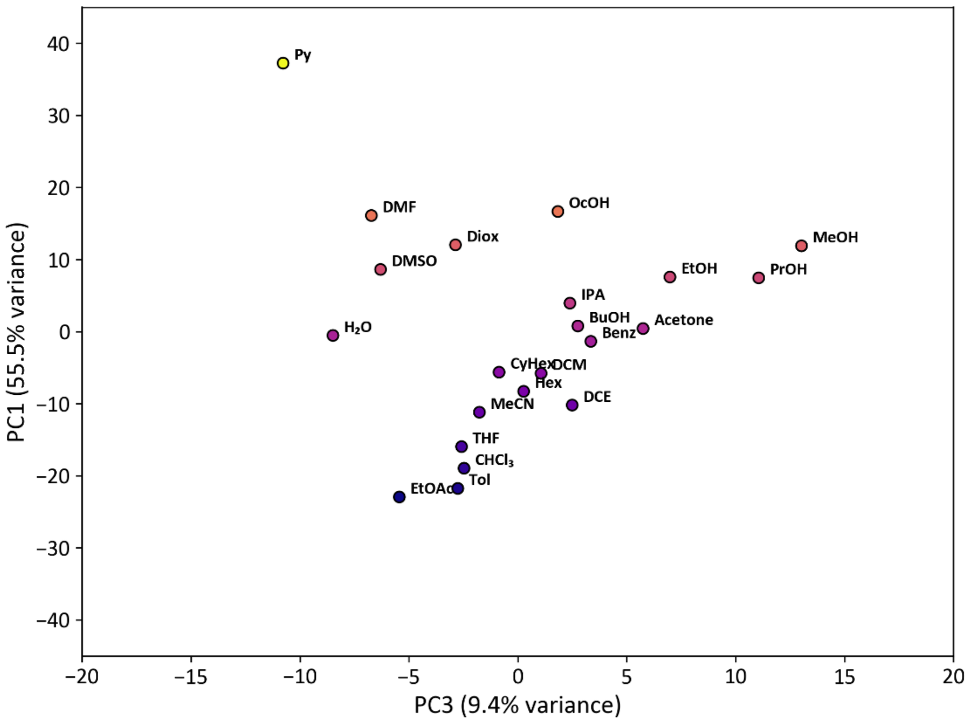

Fig. S10 2D principal component analysis (PCA) score plot of UV/VIS spectra of the modified slide in 22 different solvents, showing PC3 versus PC1. Color coding corresponds to PC1 scores as defined in Figure S7.

5. NMR spectra of stilbazolium salt 6

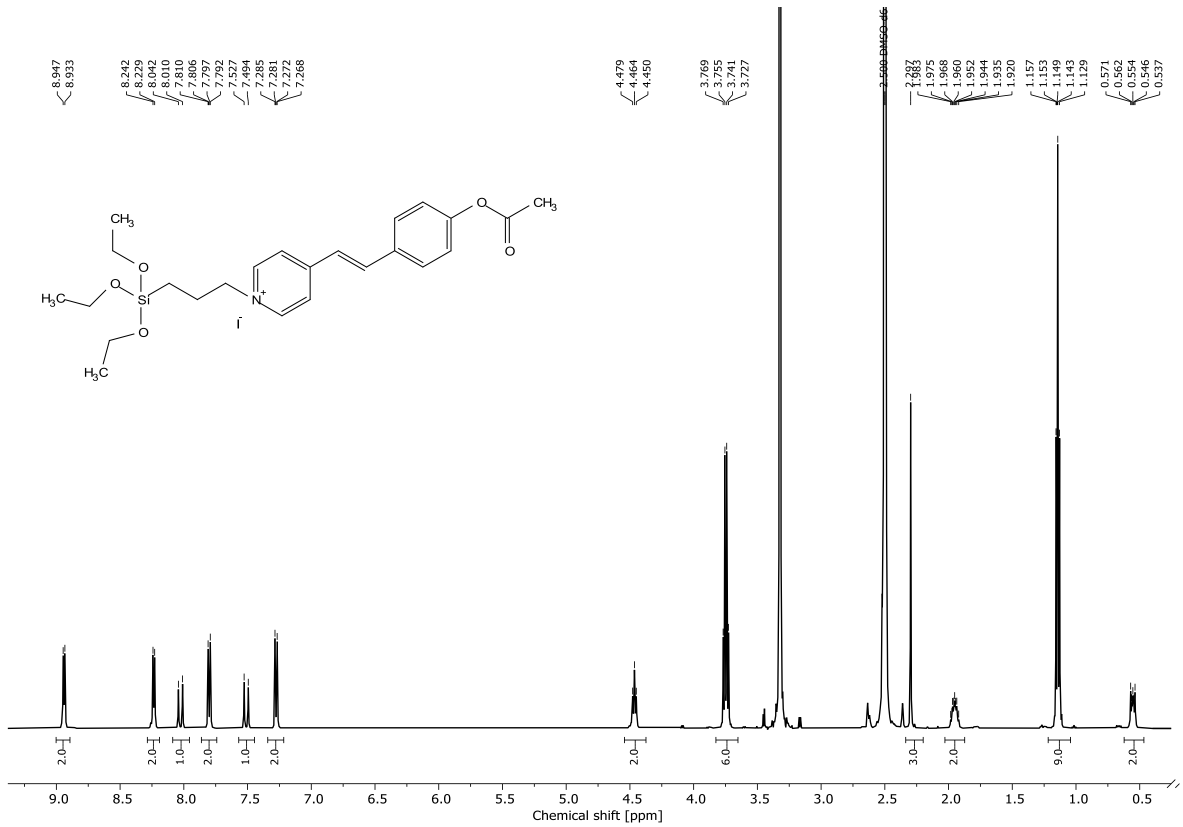

Fig. S11 ^{1}H NMR (500 MHz, DMSO-d_6, 25 °C) spectrum of stilbazolium salt **6**.

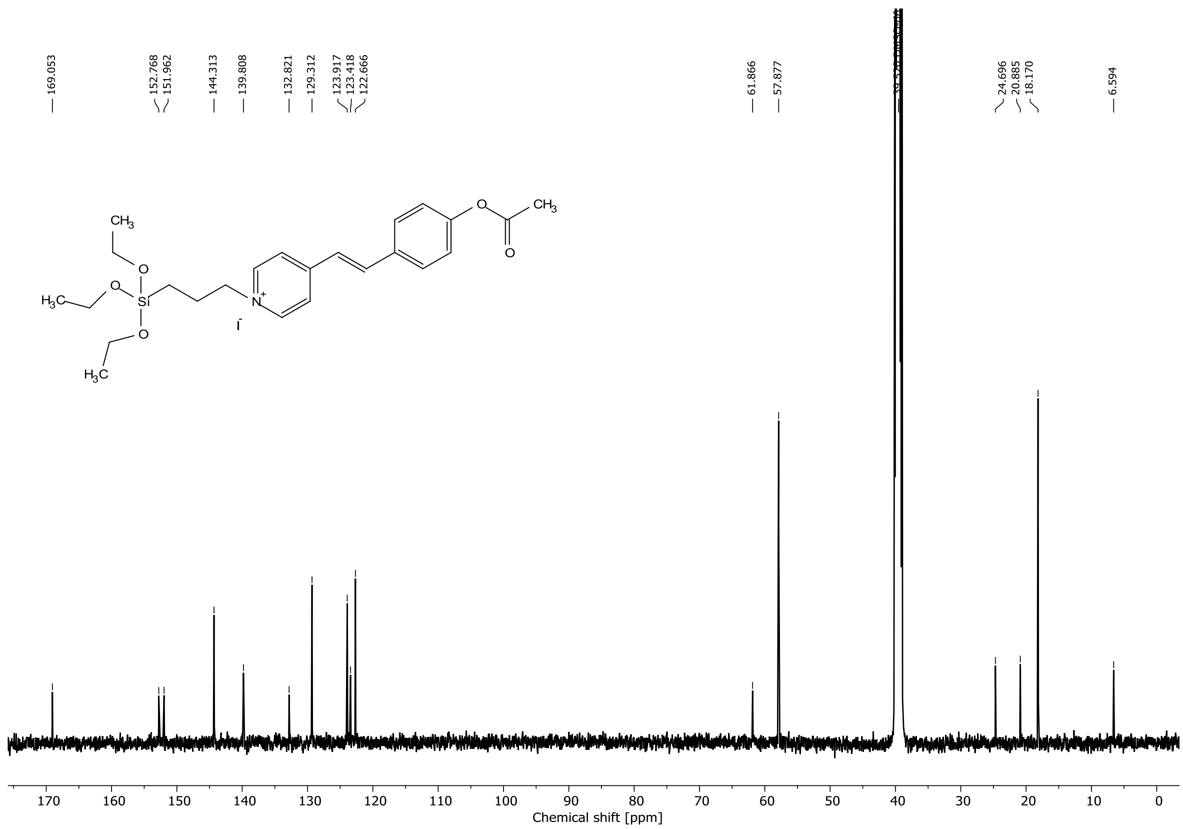

Fig. S12 $^{13}C\{^1H\}$ NMR (126 MHz, DMSO-d_6, 25 °C) spectrum of stilbazolium salt **6**.

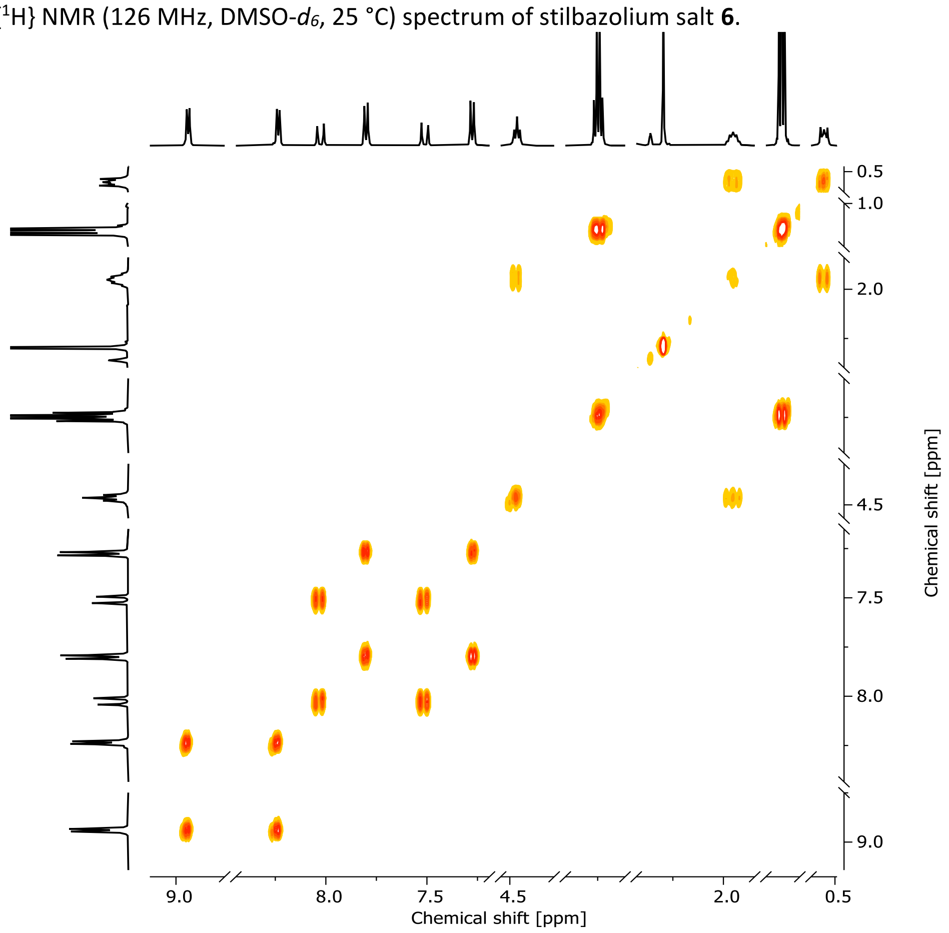

Fig. S13 Section of the ^1H-^1H COSY spectrum in the signal region of stilbazolium salt **6**.

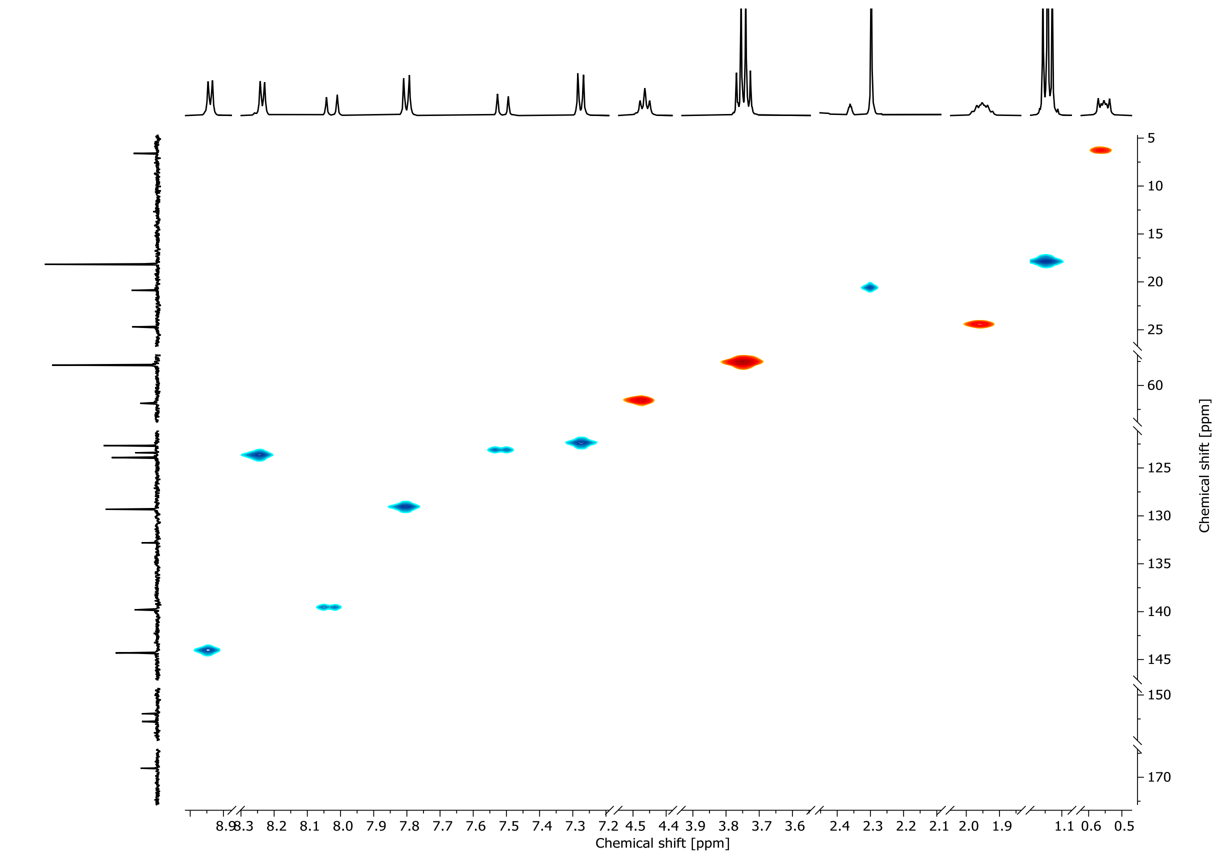

Fig. S14 Section of the ^{1}H-^{13}C HSQC spectrum in the signal region of stilbazolium salt **6**.

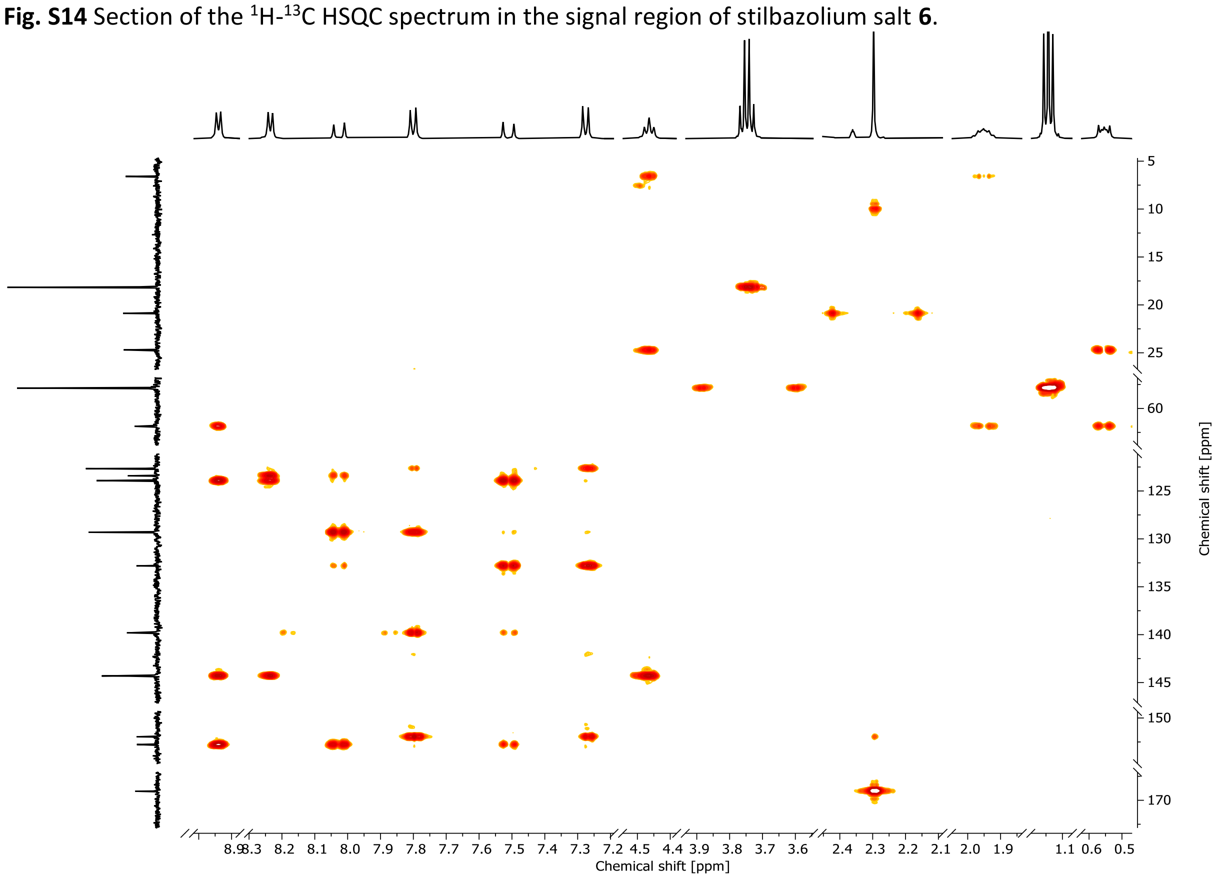

Fig. S15 Section of the ^{1}H-^{13}C HMBC spectrum in the signal region of stilbazolium salt **6**.

6. Decomposition of deacetylated stilbazolium salt 6

6.1 HRMS

HRMS (ESI$^+$, MeOH): m/z calculated for $C_{18}H_{23}NO_4Si$ $[M+H]^+$ 346.14691, found 346.14735.

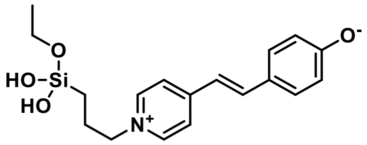

Fig. S16 Proposed structure ($C_{18}H_{23}NO_4Si$) of the decomposition product formed via deacetylation of stilbazolium salt **6**.

6.2 NMR spectra

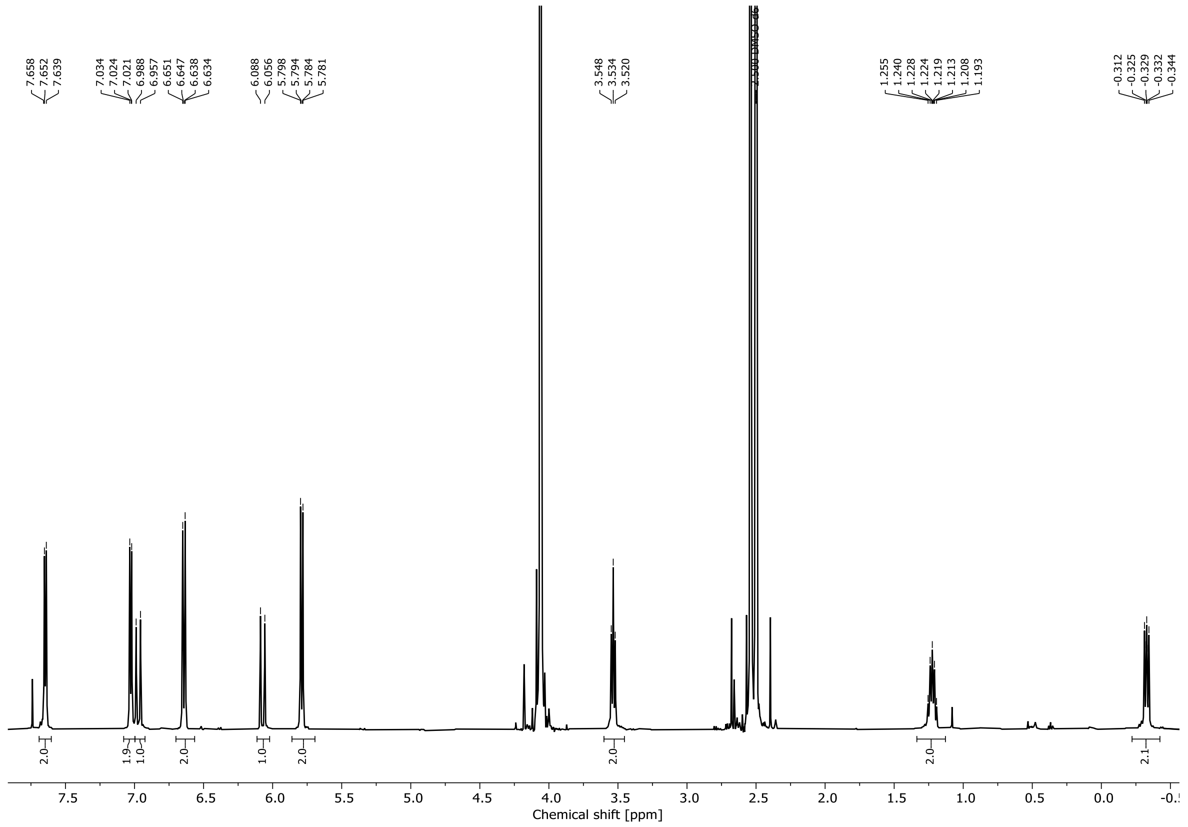

Fig. S17 ^{1}H NMR (500 MHz, DMSO-d_6, 25 °C) spectrum of decomposition product.

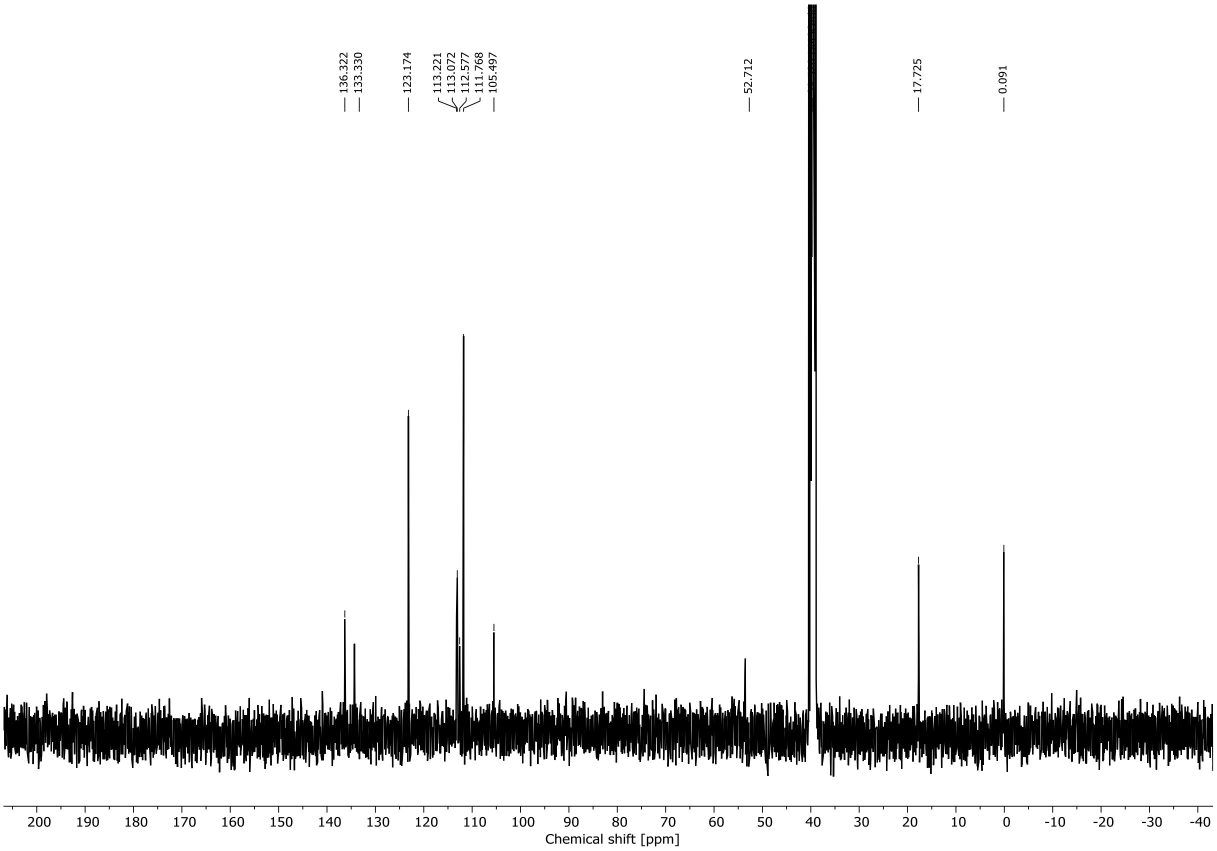

Fig. S18 $^{13}C\{^{1}H\}$ NMR (126 MHz, DMSO-d_6, 25 °C) spectrum of decomposition product.

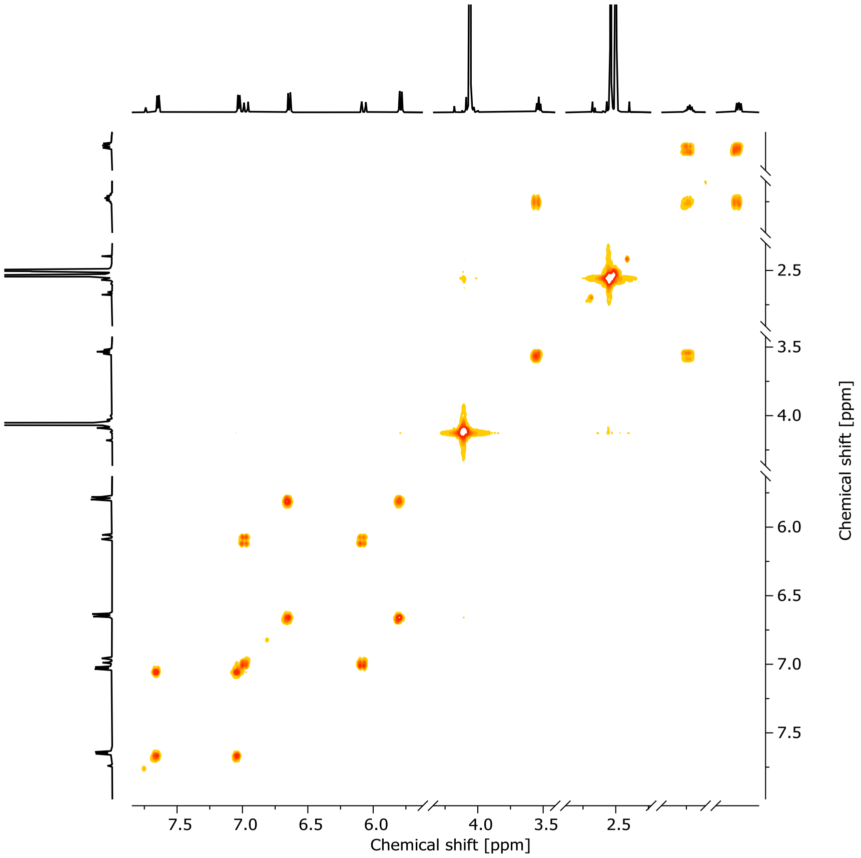

Fig. S19 Section of the ^{1}H-^{1}H COSY spectrum in the signal region of decomposition product.

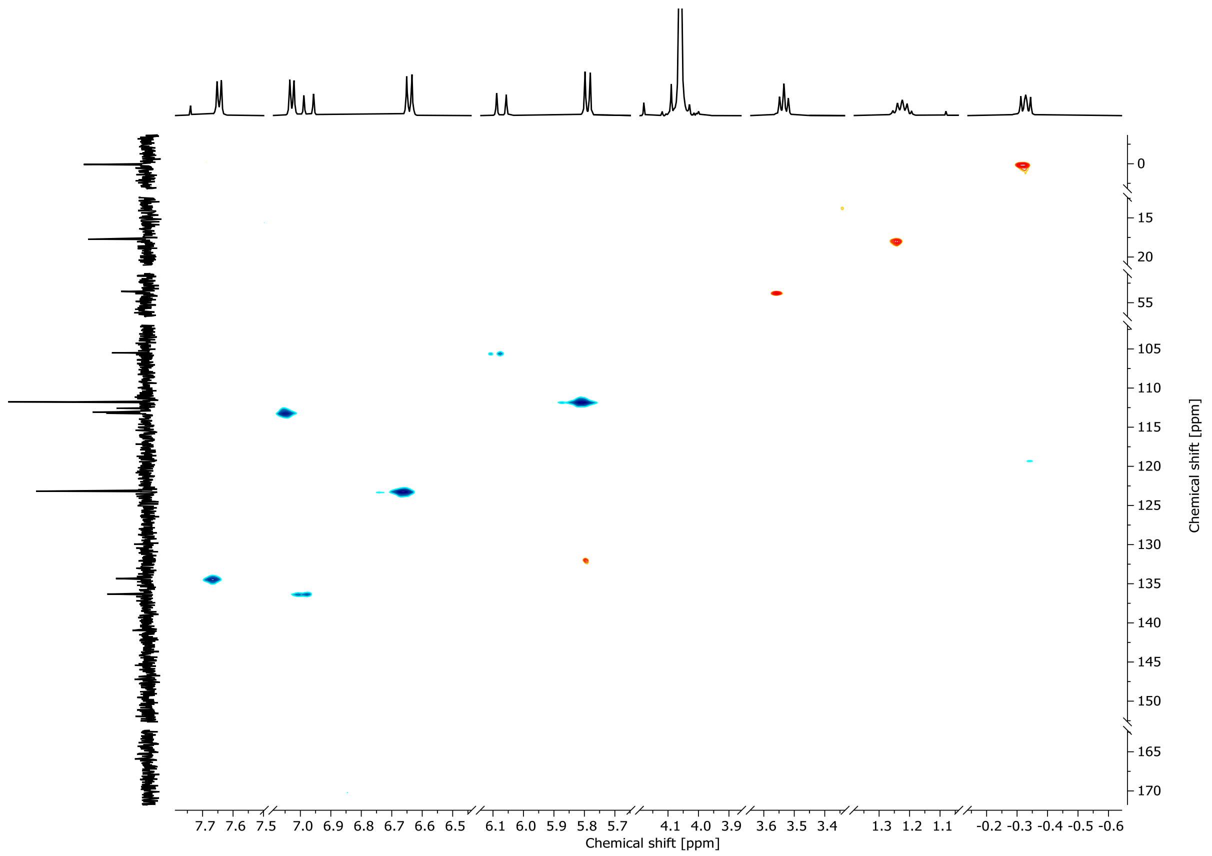

Fig. S20 Section of the ^{1}H-^{13}C HSQC spectrum in the signal region of decomposition product.

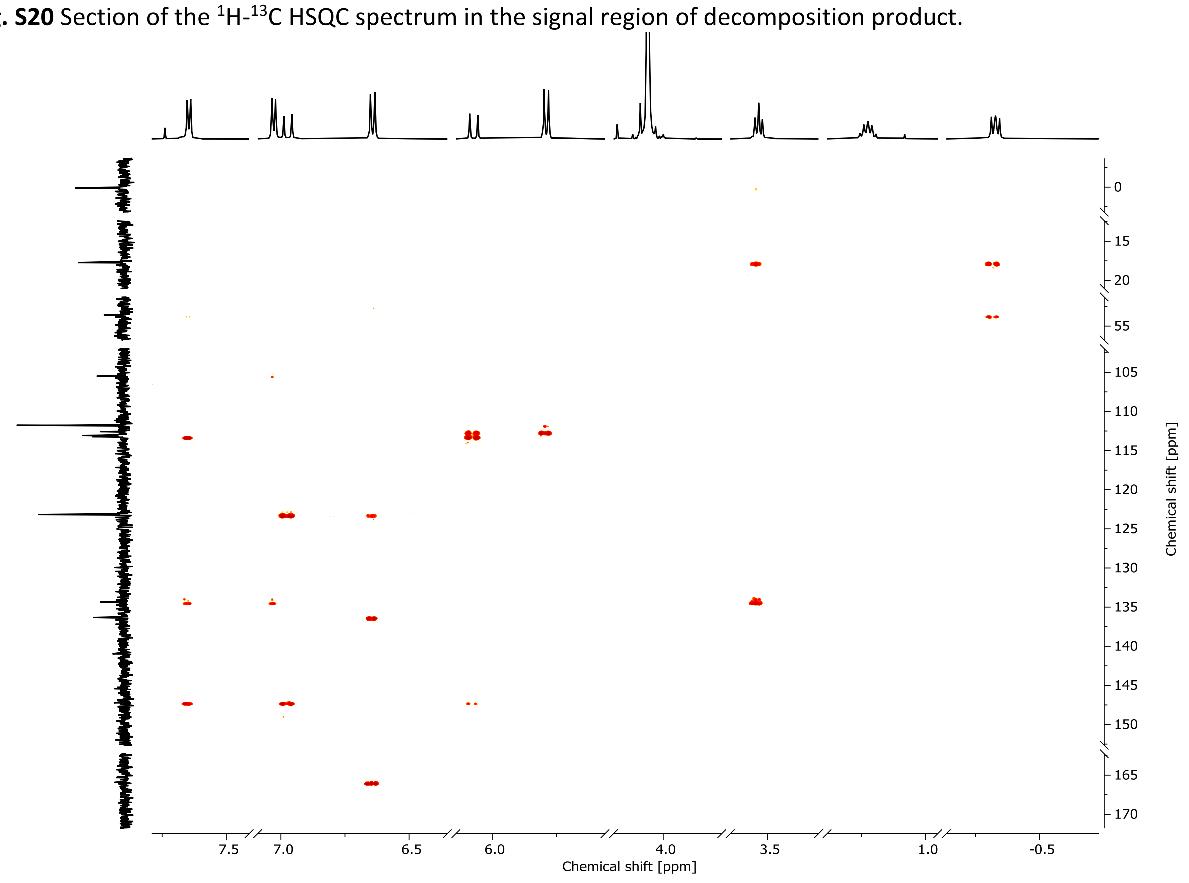

Fig. S21 Section of the ^{1}H-^{13}C HMBC spectrum in the signal region of decomposition product.
